# Perspectives on barriers to treatment engagement of people with eating disorder symptoms who have not undergone treatment: a qualitative study

**DOI:** 10.1186/s12888-022-03890-7

**Published:** 2022-04-04

**Authors:** Livia Liu, Phillipa Hay, Janet Conti

**Affiliations:** 1 Translational Health Research Institute, School of Medicine, Western Sydney University, Sydney, Australia; 2 School of Medicine, Western Sydney University, Locked Bag 1797, Penrith, NSW 2751 Australia; 3 School of Psychology, Western Sydney University, Sydney, Australia

**Keywords:** Eating disorders, Treatment barriers, Qualitative

## Abstract

**Background:**

Many people with an eating disorder (ED) never engage with an evidence-based ED treatment. Of the few studies that have qualitatively explored barriers to receiving treatment, some do so in relation to mental health conditions in general, and others focus on participants who have already undergone treatment.

This study aims to address this gap in the literature by exploring the barriers to ED treatment engagement from the perspectives of individuals in the community with an ED (either self-identified or professionally diagnosed) and had not received ED treatment/s.

**Method:**

Fifty-six of 772 participants in an online Eating Disorders Treatment Experience survey had self-identified as having symptoms consistent with an ED, or had received a diagnosis of an ED and indicated that they had never undergone treatment for an ED. They were asked to share the reasons for which they did not receive treatment with an open-ended question. Qualitative analysis of survey responses was completed using the Framework Method to generate overarching themes that encapsulated the diverse participant accounts.

**Results:**

The thematic analysis generated two main themes, each with two subthemes. The first theme was the negotiation of the need for treatment within oneself (intrapersonal factors; theme 1). The second theme explored interpersonal contexts that shaped the participant’s decision not to seek treatment (interpersonal/external factors; theme 2). Two cross-cutting subthemes of fear and health literacy were also generated that demonstrated a high degree of overlap with the aforementioned main themes.

**Conclusions:**

The process by which individuals decide whether or not to engage with ED treatment services is complex and involves intra- and interpersonal negotiations intertwined with health literacy and fear. A factor not prominent in previous research was negative self-perceptions and the belief of being undeserving of treatment. These factors have implications for ongoing community and clinical interventions to further address barriers to ED treatment engagement.

## Background

Eating disorders (EDs) include the conditions of anorexia nervosa, bulimia nervosa, binge eating disorder, avoidant/restrictive food intake disorder, other specified feeding or eating disorder and unspecified feeding or eating disorder [1]. Typically, those with an ED experience altered cognitions leading to a preoccupation with weight and body shape that drives behaviours, including restrictive eating, binging and purging. People with EDs experience physical complications, associated with impaired nutrition and decreased quality of life, as well as psychological complications, with major depressive disorder being the most common [2]. The social and economic burden of EDs is also significant, with the estimated annual cost in South Australia in 2018 being $84 billion. This included costs associated with burden of disease as the result of years lived with disability, years of life lost as well as health system costs and productivity loss [3]. The rate of mortality for EDs is 10 to 12 times greater than the general population [4]. Despite this burden [5] and high general healthcare service utilisation [6], only 23.2% of people with EDs are estimated to have sought specialist ED treatment [7] with a median delay of 10 to 15 years between the onset of symptoms and seeking help [8]. Although data regarding treatment-seeking is difficult to obtain due to lack of engagement with services, it has been reported that the rates of treatment-seeking amongst those with EDs is 19–36% less than that of other mental health problems [9].

Literature examining barriers to mental health treatment in general is relatively comprehensive and stigma, loss of confidentiality, lack of insight and accessibility have been identified as significant factors [10]. Similar issues were reported in a systematic review of thirteen studies that investigated the perceived barriers and facilitators of seeking treatment in EDs [11]. This included eight qualitative, three quantitative and two mixed-methods studies. The perceived barriers identified were stigma and shame, denial of the severity of the illness, practical barriers such as cost, low motivation, negative attitudes, lack of encouragement from others to seek help and lack of knowledge about the resources available. It was noted that many studies in the review focused only on one or a few treatment barriers. Additionally, some studies focused on specific populations (e.g. Latina women, female university students, high school females, Mexican American women in the Los Angeles area) and thus, data may not be reflective of the wider population. Another systematic review [12] examined forty-four qualitative and twenty-four quantitative papers to better understand the perspectives towards barriers and facilitators of ED treatment of those with EDs as well as family, friends and health professionals. The first dominant theme identified was ‘the help-seeking process at primary care’ that described primary care providers as frequently failing to detect ED symptoms and provide a timely diagnosis. The second theme was ‘expectations of care and appropriate referrals’ which described patient and family member perspectives of factors that constituted effective treatment pathways such as involvement of professionals with extensive experience with EDs and treatment in an environment that felt safe and supportive. The final theme was ‘opposition and collaboration in the treatment of and recovery from eating disorders’ which explored collaboration between patient and healthcare providers as a treatment facilitator and opposition and hostility as treatment barriers.

Since these reviews, one cross-sectional study [13] has further analysed data from a survey of 425 participants with a 25-item questionnaire. In this study, the strongest barriers to seeking treatment were fear of losing control, fear of change and lack of motivation. Participants also had more positive attitudes towards established treatments than towards novel ones. Whilst quantitative data provides valuable information regarding a range of attitudes and their relative significance to patients, qualitative studies facilitate in-depth exploration of the perspectives and experiences of those with a lived ED experience. In this regard, psychosocial barriers to engagement with ED treatment have been explored qualitatively with thirteen people in the United Kingdom who failed to follow-up with specialist treatment after referral from a general practitioner [14]. Barriers identified included: practical barriers such as administrative errors, long waiting times and distance from care, and complex social issues such as childhood trauma and fear of losing control. Depression and anxiety were also found to play a pertinent role in hindering the ability of participants to engage in treatment or even leave the house to attend initial appointments. This study highlighted the range and complexity of reasons why people did not seek treatment and the need for further research to understand these processes of decision-making and implications for primary and specialist treatment providers.

To our knowledge, this is the first study which has explored, with an in-depth qualitative approach, reasons for not engaging in treatment amongst a community sample who have reported never undergoing ED treatment. Ultimately, whilst barriers to ED treatment have been identified, these are mostly in studies focused on those who had already undergone, or were in the process of undergoing treatment, perhaps due to relative ease of participant recruitment. Retrospective recall bias may have influenced the findings and therefore this qualitative study aimed to explore the perceptions of a community sample of participants who report never having received treatment for an ED and the barriers that they identified. In the present study most participants had self-identified to have symptoms of an ED however had never sought diagnosis or treatment, and some had previously received a diagnosis from a primary care provider but not followed through with specialist treatment.

## Method

### Design

This study was embedded in an online survey conducted in 2018–2019 and comprised a subsample of participants who self-identified as having experienced ED symptoms though had not received treatment. They were recruited via the Eating Disorders Treatment Experience survey developed by authors (PH and JC) which was advertised online through Facebook pages in Australia, New Zealand, the United States and the United Kingdom in 2018–2019. Advertisements were targeted at people who self-identified as experiencing or having experienced an ED and linked respondents to the online survey.

Ethical approval was obtained from the Western Sydney University Human Research and Ethics Committee (HREC), (Approval number H11739) with an amendment in 2020 to add the student to the research team. Informed consent was obtained from all participants and/or their legal guardians and the research involving human research participants was performed in accordance in accordance with the relevant guidelines and regulations and with the Declaration of Helsinki.

### Participants

Seven hundred and seventy two survey respondents (708 female, 18 male, 46 non binary/prefer not to say, age range 15–74; M = 23.18, SD = 9.26) affirmed that they had experienced symptoms of an ED (for example, purging, restricting food intake). Participants in the present study were the 56 individuals who reported not receiving ED treatment due to not seeking a formal diagnosis and referral, or due to failure to follow-up after a diagnosis; i.e. they responded to the question: “Have you ever received treatment for an eating disorder?” with “No, I have never undergone treatment for an eating disorder”. Although not specifically asked, 5 of the 56 included participants noted in their responses that they had previously sought help but did not end up undergoing ED treatment. The 56 participants were majority women (*n* = 48, 85.7%), 3 were men (5.4%), and 5 non-binary/prefer not to say gender (8.9%), with ages ranging between 16 and 59 years (M = 23.28, SD = 11.40).

### Assessment

The Eating Disorders Treatment Experience survey consists of both open and closed questions of qualitative and quantitative data. The survey collected demographic and clinical data, with open-ended qualitative questions on treatment experience in addition to the following scales: the Eating Disorder Examination Questionnaire Short (EDE-QS) [15] and the Hospital Anxiety and Depression Scale (HADS) [16].

The EDE-QS is a 12-item questionnaire that assesses the range and severity of ED features in which a higher score indicates greater levels of symptoms and a score of > 15 indicates possible presence of an ED [17]. EDE-QS scores have a reported high internal consistency of α = 0.913 amongst a population of 559 individuals aged 18–74 with a history of EDs [15]. In the current study, the internal consistency was found to be α = 0.812. The HADS is a 14-item questionnaire used for screening of mood disorders and consists of an anxiety scale (HADS-A) consisting of 7 questions and separate depression scale (HADS-D) with a further 7 questions. Each scale has a maximum score of 21 with higher scores indicating more distress. The internal consistency of the anxiety subscale was reported by Zigmond and Snaith to be 0.76 to 0.41 whilst that of the depression subscale was reported at 0.60 to 0.30. In the current study, the internal consistency of the HADS was found to be α = 0.763 overall, 0.765 on the items for anxiety and 0.708 on the items for depression.

This present study focused on analysis of the data generated by the open-ended question that asked participants: “Could you let us know why you decided not to seek treatment for an eating disorder either currently or in the past?”

### Analysis

Data were analysed by identifying recurring themes, opinions and beliefs in the answers to the open survey question. The five-staged Framework Method [18], suitable for analysis of cross-sectional descriptive data [19], was utilised with an inductive approach. The framework method is based on a set of principles shared by other qualitative methods [19], such as thematic analysis [20], that includes researcher immersion in the data and noting of early impressions (stage 1, LL), and the development of a coding system and linking codes or units of data to form overarching themes (stage 2; LL with consensus reached with JC & PH). Following this was the third stage (indexing) in which a numerical code was utilised to annotate the survey responses. In stage 4 (charting), the data was rearranged systematically using a matrix based on themes previously identified and finally, stage 5 involved mapping and interpretation of the identified themes and the associations between them were explored. Exemplar quotes were analysed in-depth to highlight the complex inter-relationship between the themes. These quotes include reference to the age and ED symptom pattern of the participant. The data in stages 4 and 5 were reviewed multiple times by both LL and JC.

## Results

Clinical features of the 56 participants are shown in Table 1. Most had not received a formal ED diagnosis but the current levels of ED symptoms were high and with the mean score well above that indicating a likely disorder of clinical severity. Depression and anxiety were also prevalent.

**Table 1:** Clinical characteristics of 56 included study participants

Participant feature		
	N	%
Eating disorder (ED) diagnostic status		
Self-identified to have an ED	40	71.4%
Respondents diagnosed with an ED by a professional	16	28.6%
Total professional diagnoses	26^a^	
Anorexia nervosa	7	44%
Bulimia nervosa	8	50%
Binge eating disorder	5	31%
Orthorexia nervosa	1	6%
Other	5	31%
	**Range**	**Median (IQR)**
Current symptoms		
EDE-QS^b^ Global Score	8–30	24 (6.25)
Days with deliberate limiting of food in past week	0 to 6–7	6–7 (1–2 to 6–7)
Days with purging or laxative use in past week	0 to6–7	0 (0 to 1–2)
Days with compulsive exercising in past week	0 to 6–7	1–2 (0 to 3–5)
Days with sense of losing control over eating in past week	0 to 6–7	3–5 (1–2 to 6–7)
Days with binge eating in past week	0 to 6–7	1–2 (0 to 3–5)
HADS^c^- depression	3–19	10.5 (2.5)
HADS - anxiety	4–21	14.5 (5)

^a^6 reported more than one diagnosis

^b^*EDE-Q* Eating Disorder Questionnaire Short 12-item

^c^*HADS* Hospital Anxiety and Depression Scale

The thematic analysis exploring participant reasons for not seeking treatment generated two main themes, namely

Theme 1: Negotiation of the need for treatment within oneself (intrapersonal factors); and

Theme 2: Negotiations of the need for treatment within a social and interpersonal context (interpersonal/external factors).

**Fig. 1:**
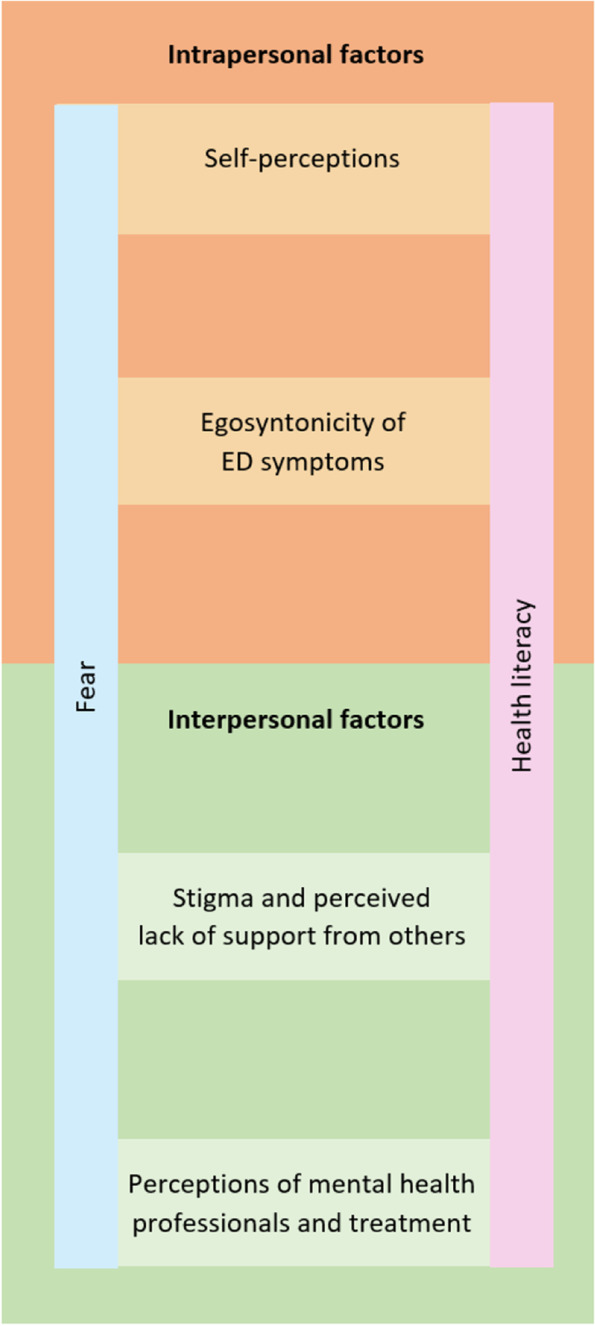
Thematic Map of Barriers to ED Treatment Seeking and Engagement

Intrapersonal factors identified as contributors to non-treatment seeking and engagement included the subthemes of self-perceptions and the egosyntonicity of ED symptoms. On the other hand, interpersonal/external factors that contributed to non-treatment seeking and treatment engagement included the subthemes of stigma and perceived lack of support by others, and perceptions of mental health professionals and treatment. Extending across both of these main themes were the cross-cutting subthemes of the emotion of fear and the concept of health literacy. Health literacy is defined as skills that enable individuals to make appropriate health decisions and successfully navigate the healthcare system [21]. In this analysis, we explore the impact of the health literacy of both respondents and healthcare providers in relation to ED treatment. Figure 1 depicts the dynamic inter-relationship between themes, subthemes and the two cross-cutting subthemes. Despite distinct themes being constructed through the thematic analysis, a high degree of overlap existed between the themes with many respondents shifting between intrapersonal and interpersonal factors in their responses, thereby constructing the sense that barriers to treatment seeking are multifactorial, interrelated and complex.

### Theme 1: Intrapersonal factors

The first theme generated from the thematic analysis involved the processes by which participants negotiated within themselves about whether or not to undergo treatment. Some respondents named the cross-cutting subtheme of fear in their responses, and one participant simply stated “fear” (non-binary, 17, restricting, excessive exercise) as the reason for not engaging in ED treatment services. Within these brief responses, the contributing factors of fear were obscured. More detailed written responses revealed how the cross-cutting subthemes of fear and health literacy intersected with the two subthemes of theme 1 that were self-perceptions and the egosyntonicity of ED symptoms.

#### Negative self-perceptions

An impoverished self-image was evident in reasons cited by participants in not progressing into ED treatment. For example, two participants (3.57%) attributed reasons for not seeking treatment to negative views of themselves with one of the few male participants stating “I don’t deserve it” (male, 17, restricting, purging or laxative use, excessive exercise). Another female participant expressed the belief that she was “too fat to deserve help” (female, 16, restricting, purging or laxative use, excessive exercise, loss of control over eating), reflecting an internalised weight stigma that contributed to her perceived unworthiness of treatment. Implicit in this statement is the assumption that a low BMI is a prerequisite to qualify for ED treatments.

In five (8.93%) of the responses, negotiations about whether or not to seek treatment were centred upon questions of health literacy including the belief of oneself as “not sick enough” (female, 20, restricting, purging or laxative use, excessive exercise, loss of control over eating, binge eating) and “not underweight” enough (female, age not specified, restricting, loss of control over eating) to receive treatment. Therefore, a low weight for these participants signified both the presence of illness and, following on from this, the sense of oneself as deserving of treatment for an ED. The following extract exemplifies the shifting nature of these negotiations as this participant alternates between minimisation of symptoms/the need for treatment, and the recognition of experiencing “a lot of worrisome thoughts about my body shape, weight, and food intake”.

EXTRACTS 1

*I’m not sick enough- I’m barely underweight, I aim to binge and purge fewer than 6 times a month, many days I eat to a slight calorie deficit. Although I have a lot of worrisome thoughts about my body shape, weight, and food intake, I also have many things I enjoy and responsibilities I don’t want to put on hold to get treatment. I will get treatment when I am emotionally and mentally completely committed to recovery, and at this point I don’t feel like I am that unhealthy or that I can get over the fear of gaining weight or losing control to really recover.*

*(female, 20, restricting, purging or laxative use, excessive exercise, loss of control over eating, binge eating)*

This participant’s account illustrates how the cross-cutting subthemes of both fear (“worrisome thoughts”) and health literacy (“I’m not sick enough”) underpin these negotiations where the decision not to seek treatment was given momentum by the sense that she would need to put her life “on hold to get treatment” and the egosyntonicity of the ED symptom that equated recovery with “losing control”.

#### Egosyntonicity of ED symptoms

The egosyntonic nature of ED symptoms was expressed in 11 (19.64%) of the responses as a reason not to seek or engage in treatment. Recovery in these contexts was considered undesirable for example, “(I) don’t want to recover” (non-binary, age not specified, restricting, purging or laxative use, excessive exercise). These participant accounts indicated an investment in maintaining the ED symptoms as evident by expressions of a commitment to “continue losing weight” (female, 18, restricting, excessive exercise, loss of control over eating, binge eating) and of the notion “Why seek treatment if it will make you fatter?” (female, 32, restricting, excessive exercise, loss of control over eating). A further restraint to treatment-seeking was an identity investment in the ED, as exemplified by another respondent who stated she did not want to “lose my eating disorder… if I’d get help it would be taken away from me” (female, 17, restricting, excessive exercise). This indicates that for this participant, treatment was associated with a loss of the ED identity and a sense of control over ED symptoms, which she was not willing to consider at the time of her response to the survey.

The cross-cutting subtheme of fear was evident in the egosyntonicity of the ED symptoms, acting as a further barrier to the utilisation of ED treatment services. This manifested as a “fear of gaining weight or losing control” (female, 20, restricting, purging or laxative use, excessive exercise, loss of control over eating, binge eating).

Similarly, there was intersection between the cross-cutting subtheme of health literacy and the egosyntonicity of ED symptoms with these participants expressing that they did not “see it as such a big deal” (female, 17, restricting, purging or laxative use, excessive exercise, loss of control over eating, binge eating). This stated reason for not utilising ED treatment services minimised the ED symptoms, reflecting an egosyntonicity, and demonstrated an ambivalence about the importance of treatment with an underlying fear and absence of a vision of who they might be if they took steps towards treatment and change.

### Theme 2: Interpersonal/external factors

The second theme of interpersonal/external factors related to participants’ perceptions of their relationships with others and their external environment and how this shaped their decision of whether or not to engage in ED treatment. The subthemes of stigma and a perceived lack of support from others, and perceptions of mental health professionals and treatment were identified and the overlap between these subthemes and the cross-cutting themes of fear and health literacy were also explored.

#### Stigma and perceived lack of support from others

In fifteen (26.79%) responses, stigma and/or a perceived lack of support from others were identified as a contributing factors to participants’ lack of engagement with ED treatments. In particular, responses indicated an avoidance of disclosure of the ED to others due to fear (cross-cutting theme) about how others would perceive them, for example that they would be “seen as weak or be treated differently”. Alongside stigma was the experience of “non-supportive people in my life” (female, 19, restricting, loss of control over eating, binge eating), where an absence of health literacy (second cross-cutting theme) and possible stigma were implicit as they recounted their perceptions of others’ minimisation of their ED experience. Examples of these perceptions of their family’s response to getting treatment for the ED included:

*“My family didn’t want me to” (female, 16, restricting, loss of control over eating, binge eating) indicating possible stigma related to having a child with an ED; and*

*“My family thinks I’m just faking this” (female, 17, restricting, purging or laxative use, loss of control over eating, binge eating) indicating a minimisation of the seriousness of an ED (health literacy).*

For these participants, familial and parental support for treatment-seeking is more relevant as they are minors whereas this did not emerge as a treatment barrier amongst adult participants. Furthermore, the fear of stigma as experienced across diverse contexts acted as a powerful restraint against treatment-seeking in both these accounts.

EXTRACTS 2

*Extract 2a*

*I'm so afraid of what it will do to my record in general of having a future (medical/mental health history when looking for a job etc.) and I'm afraid of the judgement I'll receive. I just really am not sure my town is very good with eating disorders based on what friends with an ED has told me.*

*(female, 17, restricting, purging or laxative use, excessive exercise, loss of control over eating, binge eating)*

*Extract 2b*

*I considered seeking help previously & even went to see a psychologist, but then when I was there I felt too scared to bring it up. None of my family knows about my eating disorder as I've been hiding it for years, I'm scared they would find out if i were to get treatment & they would be disappointed. I also felt that I would not be able to get treatment or be diagnosed properly due to the fact that I'm not skinny enough still, even though I have had disordered eating habits & thoughts etc for years*

*(female, 19, restricting, purging or laxative use, excessive exercise, loss of control over eating)*

Extract 2a exemplifies the fear of the stigma of an ED diagnosis and fear of the material effects of not being given equal opportunities in the workplace because of an ED history. A perception of stigma was also implicit in Extract 2b in the participant’s fear of family disappointment. Furthermore, in both these extracts were the participants’ beliefs that treatment programs available to them did not have sufficient health literacy to understand and treat EDs with the second participant expressing concern that, in the absence of being “skinny enough”, there existed a risk of misdiagnosis and exclusion from treatment.

Some participants mentioned difficulties in communicating the ED experiences with others, for example “(I) don’t know how to tell people I’m having issues” (female, 16, restricting, purging or laxative use, excessive exercise, loss of control over eating, binge eating).

#### Perceptions of mental health professionals and treatment

Nearly half the respondents (*n* = 24; 42.86%) cited treatment access and/or their perceptions of mental health professionals as reasons why they did not seek or progress with treatment. For those who had sought treatment in the past, negative treatment experiences also acted as a barrier to pursuing treatment, including for example, “Because I went to the therapist for other reasons and hated it, so I don’t want to seek treatment” (female, 17, restricting, excessive exercise, loss of control over eating, binge eating). Other participants described difficulties in accessing appropriate services because it was “unknown to (her) what services” were available (female, 21, restricting, excessive exercise, loss of control over eating, binge eating). Another reported multiple referrals “three different services (GP, eating disorder clinic, community mental health)” (female, 41, loss of control over eating, binge eating) indicating that for some participants, barriers to treatment seeking were structural and related to a lack understanding of available services and pathways of referral amongst healthcare professionals.

Financial access barriers were prominent and not having “funds to seek treatment” (female, age not specified, restricting, loss of control over eating). Geographical distance due to a lack of local services was also noted, with one of the two male participants describing a scarcity “in a small rural town away from intensive treatment” (male, 19, restricting, loss of control over eating). Implicit in this is perhaps a perception that the sacrifices associated with treatment-seeking when access is difficult outweigh the benefits offered by engaging with healthcare services. Furthermore, the same respondent expressed the perception that he would “not fit in with the other patients”, suggesting that barriers associated with access was not the sole factor contributing to the decision to not seek treatment and potentially reflecting the notion that barriers to ED treatment-seeking for men are also gendered. This is supported by Thapliyal et al. [22] who reported gendered constructions that shaped perceptions of what it meant to be a male seeking ED treatment and the need for developing tailored interventions for men with EDs as many men felt that their needs were not met by the treatment options available.

Cutting across this subtheme was a fear of how health professionals might respond if treatment was sought which was underpinned by an apparent lack of trust in the competency of health professionals in treating EDs.

EXTRACTS 3

*Because I am already considered overweight I was utterly scared to voice my feelings and actions with any doctor or professional. As I was scared that they would say “well there’s nothing to worry about “ as I’m not in immediate physical danger*

*(female, 19, restricting, loss of control over eating, binge eating)*

*I’m worried doctors will think I’m making it up or won’t take me seriously*

*(female, 17, restricting, loss of control over eating).*

*It's only when you're clearly underweight and have fainted in public that people notice*

*(female, 21, restricting, excessive exercise, loss of control over eating, binge eating).*

*I haven't spoken to anyone who has listened or saw through what I was saying about my weight*

*(female, 17, restricting, loss of control over eating, binge eating).*

These extracts exemplify healthcare mistrust amongst participants and a perception that if they were to seek treatment that health professionals would lack the capacity to identify eating disorders or take them “seriously”, unless they were underweight and the health consequences of this were clear (e.g. “fainted in public”). This perception reflects gaps in the health literacy of these participants. The invisibility of the ED symptom for one participant contributed to a lack of trust that health professionals would listen enough to see “through” what she was saying.

Fear of engaging with a health professional was evident in these extracts, namely fear that their experiences would be minimised and/or that they would not be understood, should they seek treatment. This also seemed to be embedded in mistrust of ED treatment services. Additionally, a fear of treatment itself was evident. For example, one participant expressed fear of the unknown regarding treatment: “I’m also afraid of what treatment could entail so I think that’s part of my avoidance” (female, age not specified, restricting, loss of control over eating). Another participant, who identified as non-binary, reported having been offered inpatient treatment in the past but “was too scared at the time to be admitted into hospital” (non-binary, 21, restricting, excessive exercise, loss of control over eating, binge eating). This could be reflective of the additional barriers faced by those who identify as non-binary in mainstream ED services [23].

EXTRACT 4

*I don’t think it is a huge problem, I am just losing weight, who never wants to? If I go to the doctor to be diagnosed or seek treatment, it will become a REAL problem, but until that (which will probably never happen), it’s nothing more than a weight loss*

*(female, 17, restricting, excessive exercise, loss of control over eating)*

This participant minimised her experience as “nothing more than a weight loss”, reflecting the notion that weight loss in a weight stigmatising society can be seemingly positive. In her response, diagnosis and treatment-seeking was positioned as problematic (“it will become a REAL problem”) and something that she was not ready to face. Implicit in this response is that for this participant, in the absence of a diagnosis and treatment, she could sustain the position that normalised her ED symptoms and circularly justified the lack of need for treatment.

## Discussion

The present study explored attitudes and beliefs towards engaging in ED treatment amongst people who had not yet received treatment. Two main themes were identified in the qualitative analysis, with the first theme encompassing the intrapersonal aspects that impeded on treatment-seeking and engagement and included the subthemes of self-perceptions and the egosyntonicity of ED symptoms. Individuals perceived themselves to be undeserving of treatment due to negative self-image, or not unwell enough to warrant treatment, and the egosyntonicity of ED symptoms manifested primarily as a fear of the unknown and a lack of desire for recovery. Conversely, theme 2 focused on the factors in participants’ external environments and interpersonal relationships that shaped their decision to not engage in treatment, including stigma, a perceived lack of support from others and perceptions of mental health professionals and treatment. Cross-cutting across these two main themes were experiences of fear and difficulties with health literacy that worked to mobilise decision-making processes for participants and contributing further to barriers to treatment seeking and utilisation.

Although intrapersonal and interpersonal/external factors were analysed as distinct themes, the two were interrelated and responses exhibited a high degree of overlap, reflecting the multifactorial and complex nature of factors that impede on engagement with ED treatment. This is evident in responses in which participants positioned themselves as for example being not “skinny enough” which contributed to a fear of having their ED experiences not taken seriously by healthcare professionals; the interplay between the intrapersonal factor of poor self-image and the interpersonal factor of fear of having their ED minimised by others was also influenced by a struggle with health literacy and the perception of a low BMI being required for ED treatment. This complexity reaffirms that although distinct factors can be identified, the relationship between them and the accumulation of multiple factors is what ultimately culminates in an underutilisation of ED treatment. The cross-cutting themes of fear and health literacy that are evident across both the intrapersonal and interpersonal factors and the dynamic nature of the analysis is a novel finding that aims to reflect the complexity of individuals’ relationship with ED treatment utilisation. Dr. Jennifer Gaudiani’s book ‘Sick Enough’ [24] is a resource accessible to those with EDs and their family members aiming to educate readers about the medical complications associated with EDs to challenge the common misconception of not being unwell enough to seek treatment. Such resources are valuable in increasing the health literacy of patients and those around them, and addressing the aforementioned intrapersonal and interpersonal factors that stand barrier to treatment engagement.

Some treatment barriers identified in this study reaffirmed findings of previous quantitative studies and systematic reviews. A “fear of losing control, fear of change, and finding motivation to change” were identified as the strongest treatment barriers in Griffiths’ quantitative analysis of barriers to ED treatment utilisation [13]. Whilst the current qualitative analysis did not compare the relative prevalence of different factors, similar themes were reaffirmed in participant responses. In comparing the differences between those with diagnosed and undiagnosed EDs, Griffiths et al. also found that health literacy was a stronger barrier amongst undiagnosed participants. This was reflected amongst our participants, many of whom had not received a formal ED diagnosis due to lack of engagement with mental health services. Similarly, our findings are consistent with those of the Ali et al.’s 2017 systematic review i.e. with prominent themes of stigma and shame, denial and failure to perceive the severity of the illness, practical barriers, low motivation to change and negative attitudes towards treatment, all of which emerged in our thematic analysis. Although another systematic review [10] reported similar themes, the themes of concerns for confidentiality and the preference for self-reliance did not emerge in our analysis. This may be due to the larger number of participants, with the included studies ranging from three to 3746 participants, and thus a higher likelihood of reaching saturation. Notably, although most themes identified in our thematic analysis had been reflected in previous papers, negative self-perceptions and the belief of being undeserving of treatment were not prominent in earlier research.

The strengths of this study lie in the approach used. Utilising online platforms for recruitment enabled advertisements to reach a large audience and subsequently, a large sample size was able to be obtained. Additionally, the advertising platform on mainstream social media aided in the recruitment of individuals who had not previously utilised ED treatment, most of whom had not received a formal ED diagnosis and had not engaged with mental health services. As many participants had self-identified as having an ED, the inclusion of the EDE-QS strengthened the study as ED behaviours amongst participants were able to be identified. Additionally, the inclusion of the HADS highlighted the significance of anxiety and depression as comorbid conditions. The question asked in the survey, “Could you let us know why you decided not to seek treatment for an eating disorder either currently or in the past?” was open-ended, participants were provided with the opportunity to express depth in their responses and explore the concepts they believed to be important, allowing for emergence of more themes. Additionally, data were reviewed multiple times by two authors (LL and JC) to identify as many themes as possible and to reach consensus regarding the final thematic map allowing for a more robust analysis and higher confidence in the study’s findings.

A limitation of the present study was in the wording of the question from which responses were derived for the thematic analysis, as individuals who indicated that they had not received treatment for an ED were assumed to have not sought treatment. A few participants expressed this to be confusing as although they had not received treatment, they had actually previously sought it. The data collected was part of a larger online survey in which those who indicated that they had not sought treatment were immediately redirected to the open-ended question our analysis was based on and were not prompted to answer the detailed demographic questions. Thus, we were unable to describe details such as nationality, country of residence, marital status, employment status and education level. Although the method of data collection through a survey allowed for a greater number of participants, responses were less in-depth than if an interview method was utilised where the interviewer is able to facilitate elaboration with prompts, and also seek clarification. Additionally, we are unable to ascertain if saturation was reached. Whilst ED scoring measures were used, the absence of formal ED diagnoses amongst many participants was a further limitation. It is possible some participants had subthreshold EDs where the imperative to seek help is less.

The low rates of ED treatment utilisation reported by Hart et al. (2011) and the long delays (up to 15 years) [8] between the onset of symptoms and seeking help renders this qualitative analysis clinically relevant. In understanding the barriers that prevent engagement with mental health treatment, clinicians will be better equipped to promote greater utilisation of services. Some respondents described an absence of recognition of the ED by others, reaffirming that primary care practitioners should be vigilant in looking for ED signs and symptoms especially in high risk groups as their presentation may be subtle and difficult to identify. Clinicians may also need to increase focus on the extent to which those with a living with an ED experience negative self-perceptions and how this plays a key role in their accessing of treatment services. Clinicians have an important role in developing and implementing therapeutic interventions through a strong therapeutic alliance that works to improve the person’s sense of self-worth, including the sense that they are deserving of treatment.

Future research with interventions aimed at informing healthcare professionals and increasing rates of treatment utilisation is necessary to further optimise treatment outcomes. This may involve exploring varying perspectives of ED treatment-seeking including that of primary care practitioners, mental health professionals and the families of those affected by EDs. It would also be valuable for further research to distinguish between those who had never sought treatment and those who had sought treatment but not engaged with the services to better understand the differences between the groups. Additionally, it would be interesting to further explore the impact of demographic factors such as ethnicity and age on engagement with ED treatment and in particular, the role of family perceptions in the help-seeking behaviour of minors.

## Conclusion

The participants of this study shared the commonality of having not engaged in treatment for their ED; however, the reasons for this were varied and complex. The intrapersonal factors of self-perceptions and the egosyntonicity of ED symptoms influenced the ways in which these individuals negotiated the need for treatment within themselves; additionally, the interpersonal/external factors of stigma and a perceived lack of support from others, and perceptions of mental health professionals and treatment held significance in individuals’ negotiations with those around them and their context. This study is novel as it examines barriers to ED treatment from the perspectives of individuals who have not previously undergone treatment. In further understanding the barriers to ED treatment utilisation, reform can be implemented to address such barriers, increasing engagement with services and ultimately improving treatment outcomes.

## Data Availability

The datasets used and analysed during the current study are available from the corresponding author on reasonable request.

## Abbreviations

ED: Eating disorder
EDE-QS: Eating Disorder Examination Questionnaire Short
HADS: Hospital Anxiety and Depression Scale
HADS-A: Hospital Anxiety and Depression Scale Anxiety Subscale
HADS-D: Hospital Anxiety and Depression Scale Depression Subscale
